# Ernährungsempfehlungen für Menschen mit Diabetes (Update 2026)

**DOI:** 10.1007/s00508-025-02676-7

**Published:** 2026-04-30

**Authors:** Carmen Klammer, Karin Schindler, Rita Bugl, Dagmar Plazek, Miriam Vötter, Tanja Kirchner, Claudia Martino, Jasmin Klammer-Martin, Joakim Huber, Eva-Christina Krzizek, Christina Rabl, Sabine Dämon, Friedrich Hoppichler, Alexandra Kautzky-Willer, Renate Kruschitz, Hermann Toplak, Martin Clodi, Bernhard Ludvik

**Affiliations:** 1 Abteilung für Innere Medizin, Konventhospital der Barmherzigen Brüder Linz, Linz, Österreich; 2 ICMR – Institute of Cardiovascular and Metabolic Research, Johannes Kepler Universität Linz, Linz, Österreich; 3 Klinische Abteilung für Endokrinologie und Stoffwechsel, Universitätsklinik für Innere Medizin III, Medizinische Universität Wien, Wien, Österreich; 4 Wiener Gesundheitsverband, Klinik Ottakring, Wien, Österreich; 5 Landesklinikum Melk, Melk, Österreich; 6 Bezirkskrankenhaus Schwaz, Schwaz, Österreich; 7 Österreichische Gesundheitskasse Mein Peterhof Baden, Baden, Österreich; 8 Österreichische Gesundheitskasse Mein Gesundheitszentrum Floridsdorf, Wien, Österreich; 9 Ernährungsberatung Klammer, Enns, Österreich; 10 Medizinische Abteilung mit Diabetologie, Endokrinologie und Nephrologie, Diabeteszentrum Wienerberg, Klinik Landstraße, Wien, Österreich; 11 Medizinische Abteilung mit Diabetologie, Endokrinologie und Nephrologie, Klinik Landstraße, Wien, Österreich; 12 SIPCAN – Initiative für ein gesundes Leben, Special Institute for Preventive Cardiology and Nutrition, Elsbethen/Salzburg, Österreich; 13 Abteilung für Innere Medizin, Krankenhaus der Barmherzigen Brüder Salzburg, Salzburg, Österreich; 14 Gender Medicine Unit, Klinische Abteilung für Endokrinologie und Stoffwechsel, Universitätsklinik für Innere Medizin III, Medizinische Universität Wien, Wien, Österreich; 15 Abteilung für Innere Medizin, Krankenhaus der Elisabethinen, Klagenfurt, Österreich; 16 FA für Innere Medizin, Endokrinologie und Diabetologie, Graz, Österreich

**Keywords:** Diabetes mellitus Typ 2, Ernährungstherapie, Lebensmittelbasierte Empfehlungen, Ernährungsmuster, Ernährungsstruktur, Diabetes mellitus, type 2, Nutrition therapy, Food-based recommendations, Nutrition pattern, Nutrition structure

## Abstract

Je nach Diabetesform und -therapie sollen alle Menschen mit Diabetes eine individuelle ernährungsmedizinische Beratung und Schulung durch Fachpersonal erhalten. Im Vordergrund steht weiterhin eine patient:innenzentrierte, individualisierte Beratung, angepasst an die jeweiligen Bedürfnisse und Lebensumstände der Menschen mit Diabetes. Ziel ist es, neben der Unterstützung zur Umsetzung einer ausgewogenen Ernährung gemeinsam realistische Stoffwechsel- und Gewichtsziele zu definieren, um den Krankheitsverlauf positiv zu beeinflussen und Spätfolgen zu vermeiden. Dabei sollten vor allem praxisbezogene Empfehlungen ausgesprochen werden, die persönliche Nahrungsmittelpräferenzen berücksichtigen und Hilfsmittel zur Planung von geeigneten Portionsgrößen sowie ausgewogener Mahlzeitenzusammenstellung einbeziehen. Entsprechend aktueller internationaler und nationaler Standards sollen Menschen mit Diabetes im Diabetesselbstmanagement unterstützt werden (DSMES) und erlernen, die postprandiale Reaktion auf Speisen und Getränke besser einschätzen und durch die geeignete Lebensmittel- und Getränkeauswahl positiv beeinflussen zu können. Alle Menschen mit Diabetes sollten regelmäßig, je nach individuellem Bedarf, die Möglichkeit haben, eine ernährungstherapeutische Beratung oder Schulung in Anspruch nehmen zu können. Diese Praxisempfehlung stellt eine Zusammenfassung der aktuellen Literatur zu ernährungsrelevanten Aspekten bei Diabetes dar.

## Abstract (en)

All people with diabetes should receive an individualized nutritional medical consultation and education provided by qualified professionals, depending on the type of diabetes and treatment. The focus is still on a patient-centered and personalized counselling, tailored to the specific needs, lifestyle and type of diabetes. The aim is to support the implementation of a balanced diet while jointly defining realistic metabolic and weight goals to positively influence the disease progression and prevent long-term complications. Particular emphasis should be placed on practical, food-based recommendations that take personal food preferences into account and include tools for planning appropriate portion sizes and a balanced meal composition. In accordance with current international and national standards, people with diabetes should have access to ongoing nutrition counselling and education. During the consultation they should be supported in self-management of their health condition and learn to be able to make a better estimation of the postprandial reaction to food and drinks and to positively influence this by an appropriate selection of foods and beverages. These practical recommendations summarize the most recent literature on nutritional aspects of diabetes.

## 1. Rolle der Ernährungstherapie

Die Ernährungstherapie ist effektiv, bringt einen Benefit und spielt eine zentrale Rolle im Diabetesmanagement. Eine ernährungstherapeutische Beratung ist mit einer Senkung des HbA_1c_ bei Betroffenen mit Typ-2-Diabetes um 0,3–2,0 % und bei Typ-1-Diabetes um 1–1,9 % assoziiert [1]. In den vergangenen Jahren hat sich der Fokus zunehmend von einer ausschließlichen makro- und mikronährstofforientierten Perspektive hin zu einem lebensmittelbasierten Ansatz verschoben, der den Verzehr nährstoffdichter Lebensmittel und die Etablierung gesundheitsförderlicher Ernährungsmuster in den Mittelpunkt stellt.

**Abb. 1:**
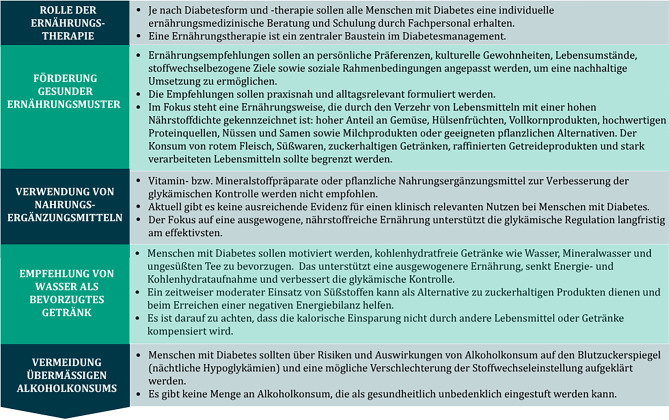
Übersicht Ernährungsempfehlungen modifiziert nach ADA 2024 [3]

Menschen konsumieren keine isolierten Nährstoffe, sondern Lebensmittel in Form von Mahlzeiten und Ernährungsmustern, die langfristig die Gesundheit beeinflussen. Daher müssen Ernährungsempfehlungen praxisnah und alltagsrelevant formuliert sein [2, 3]. Universelle „One-size-fits-all“-Empfehlungen sind nicht zielführend. Individuelle Vorlieben, kulturelle Aspekte, Stoffwechselziele, soziale Faktoren und Lebensumstände müssen berücksichtigt werden, um gemeinsam personalisierte ernährungstherapeutische Interventionen zu erarbeiten. Eine Übersicht einiger Ernährungsempfehlungen ist in Abb. 1 dargestellt.

Maßnahmen der Ernährungstherapie bei Diabetes sind [3]:

1. Förderung gesunder Ernährungsmuster: Vielfalt an nährstoffreichen Lebensmitteln in ausgewogenen Mengen, um Gewicht, Blutzucker, Blutdruck und Blutfettwerte in den Zielbereich zu bringen und das Risiko für Folgeerkrankungen zu senken.
2. Individualisierung: Anpassung der Ernährungsempfehlungen an persönliche Präferenzen, kulturelle Gewohnheiten, Gesundheitskompetenz und soziale Rahmenbedingungen, um eine nachhaltige Umsetzung zu ermöglichen.
3. Erhalt der Lebensqualität: Essen soll Genuss und Freude bereiten. Empfehlungen sollen motivierend und nicht bevormundend sein, bzw. ein vermeidendes Essverhalten soll verhindert werden.
4. Vermittlung praktikabler Strategien: Menschen mit Diabetes sollen Werkzeuge und Orientierungshilfe erhalten, die im Alltag leicht anwendbar sind (z. B. das Tellerprinzip oder einfache Portionsempfehlungen), und ggf. eine Abschätzung der Kohlenhydrate bei intensivierter Insulintherapie zur Abstimmung der prandialen Insulindosis und Selbstevaluierung postprandialer Glukoseverläufe erlernen.

## 2. Makronährstoffe

Die Makronährstoffverteilung sollte individuell basierend auf den Essgewohnheiten, Nahrungspräferenzen, der körperlichen Aktivität und den Stoffwechselzielen erfolgen. Es gibt Hinweise, dass es für Menschen mit Diabetes keine ideale prozentuale Verteilung der Makronährstoffe gibt, die allgemein gültig ist [4, 5] – auf diese wird jeweils in den folgenden Kapiteln eingegangen. Die Schlüsselstrategie in der Beratung liegt im Erreichen der glykämischen Ziele, in der Bewertung der Nährstoffaufnahme sowie in der Optimierung der Auswahl von Lebensmitteln und Etablierung ausreichend körperlicher Aktivität im Alltag.

### 2.1 Kohlenhydrate

Bei der Auswahl von kohlenhydrathaltigen Lebensmitteln steht die Qualität vor der Quantität, daher sollten nährstoffreiche Kohlenhydratquellen, die ballaststoffreich und möglichst wenig verarbeitet sind, bevorzugt werden. Bei Patient:innen mit intensivierter Insulintherapie ist es von besonderer Bedeutung, den Zusammenhang zwischen Kohlenhydrataufnahme und Insulinbedarf verständlich zu machen. Dies soll im Rahmen einer intensiven Schulung zum Kohlenhydratgehalt von Speisen in Gramm an Kohlenhydraten bzw. in Kohlenhydrateinheiten (KE) je nach Therapieform erfolgen.

#### 2.1.1 Glykämischer Index

Bei der Auswahl von kohlenhydratreichen Lebensmitteln ist neben dem Ballaststoffgehalt auch der glykämische Index (GI) bzw. die glykämische Last (GL) zu beachten. Der Einfluss von Nahrungskohlenhydraten auf die glykämische Antwort hängt von verschiedenen Faktoren wie aufgenommener Menge, Art und zellulärer Struktur, thermischer und/oder mechanischer Verarbeitung sowie gleichzeitigem Verzehr anderer Makronährstoffe ab [6]. Darüber hinaus wird die glykämische Antwort auf Nahrungsmittel auch von der Nüchternblutglukosekonzentration und dem Ausmaß der Insulinresistenz beeinflusst.

Der GI ist eine Maßzahl für die Wirksamkeit verschiedener Lebensmittel auf die Blutglukose. Seine Bestimmung erfolgt, indem die Blutzuckerkurve nach Aufnahme von 50 g Kohlenhydraten aus einem Testlebensmittel über 2 h verfolgt wird. Diese Kurve wird zu jener in Beziehung gesetzt, welche sich aus dem Konsum von 50 g Kohlenhydraten in Form von Weißbrot oder Glukose ergibt. Der GI wird in Prozent in Bezug zum Referenzlebensmittel angegeben. Daher bedeutet ein GI = 70, dass die Blutzuckerwirksamkeit des untersuchten Lebensmittels 70 % der von 50 g Weißbrot bzw. Glukose beträgt (die Fläche unter der Blutzuckerkurve ist um 30 % geringer als die von Weißbrot bzw. Glukose). Die Auswirkungen eines Lebensmittels auf den Blutglukose- und Insulinspiegel hängen sowohl von der Menge der verzehrten Kohlenhydrate als auch vom GI ab. Die alleinige Betrachtung des GI hat den Nachteil, dass er sich definitionsgemäß auf 50 g Kohlenhydrate bezieht, welche nur vereinzelt praxisbezogene Verzehrgewohnheiten widerspiegelt. Folglich entsprechen 50 g Kohlenhydrate aus Karotten einer Menge von 650 g, sodass der Verzehr einer üblichen Portion zwischen 100 und 150 g trotz des höheren GI geringe Auswirkungen auf den Blutglukosespiegel hat. Die Verzehrgewohnheiten werden im Konzept der GL berücksichtigt. Die GL errechnet sich aus dem Produkt der verwertbaren Kohlenhydratmenge pro Portion und dem GI [7]. Eine Ernährungsweise mit niedrigem GI/GL kann sich demnach positiv auf den Blutglukosespiegel auswirken, aber wahrscheinlich genau deshalb, weil vorrangig minimal verarbeitete und ballaststoffreiche Kohlenhydratquellen wie Vollkornprodukte, Hülsenfrüchte, Gemüse und Obst sowie hochwertige Proteinquellen verzehrt werden [8, 9].

### 2.2 Ballaststoffe

Der häufig beobachtete ungünstige Effekt einer stärkereichen Ernährung auf den Triglyzeridplasmaspiegel [10] kann vermieden werden, wenn die verzehrten kohlenhydratreichen Lebensmittel gleichzeitig ballaststoffreich sind. Daher sind Vollkorngetreideprodukte Weißmehlprodukten vorzuziehen. Eine tägliche Ballaststoffaufnahme von mindestens 30 g/d wird empfohlen. Dies kann durch den Konsum von Vollkorngetreide, Hülsenfrüchten, Nüssen, Samen, Gemüse und moderaten Mengen Obst erreicht werden. Höhere Aufnahmen waren mit größeren positiven Effekten assoziiert [11]. Bei einer höheren Aufnahme bzw. bei einer Steigerung der Ballaststoffaufnahme ist zu berücksichtigen, dass es zu Flatulenzen und bei zu geringer Flüssigkeitszufuhr zu Obstipation kommen kann, daher ist auf eine langsame Steigerung und eine ausreichende Flüssigkeitszufuhr zu achten [12]. In der Literatur werden positive Effekte auf die Blutglukose und Insulinresistenz sowie eine geringere Mortalität beschrieben verglichen mit einer niedrigeren Ballaststoffaufnahme [13, 14]. Die Hälfte der Ballaststoffe sollte in Form von löslichen Ballaststoffen verzehrt werden (z. B. Pektine, Inulin, Psyllium). Diese finden sich vor allem in Gemüse und Obst. Der Verzehr von Ballaststoffen in Form von natürlichen Lebensmitteln ist dem von ballaststoffreichen Nahrungsergänzungsmitteln vorzuziehen, da Lebensmittel auch Mikronährstoffe liefern [15, 16].

#### 2.2.1 Insulinresistenz und Beta-Glucan

Der im Hafer enthaltene lösliche Ballaststoff β‑Glucan zeigt in Studien positive Auswirkungen auf das Glukose- und Lipidprofil bei Personen mit Diabetes mellitus Typ 2 (T2DM). Die Aufnahme von 3 g β-Glucan pro Tag, enthalten in rund 60–70 g Haferflocken bzw. 50 g Haferkleie, kann bei positivem Wirkungsprofil für Personen mit T2DM empfohlen werden. Für jene mit Diabetes mellitus Typ 1 (T1DM) fehlen noch entsprechende Daten [17, 18].

In Studien waren natürliche Haferflocken dem Extrakt von β‑Glucan überlegen [19, 20]. Zwei- bis dreitägige, hypokalorische Hafer- bzw. Ballaststofftage sind besonders im stationären Umfeld zur Therapie einer ausgeprägten Insulinresistenz zu empfehlen. In einer Pilotstudie konnte bei zweitägigen Hafertagen mit 1100 kcal aus Haferbrei eine Reduktion der mittleren Insulindosis von 47 % erreicht werden [21]. In vitro konnte eine hemmende Wirkung auf die SGLT1-Rezeptoren, die GLUT2-Transporter, die Dipeptidylpeptidase 4 (DPP4) und die Alpha-Glukosidase beobachtet werden [21–25]. Zudem bindet β‑Glucan Gallensäuren im Darm und entzieht diese somit dem enterohepatischen Kreislauf, wodurch der Cholesterinspiegel gesenkt werden kann [26, 27]. Hafertage sollten immer unter ärztlicher und ernährungstherapeutischer Begleitung stattfinden.

### 2.3 Fett

Der Anteil der täglich aufgenommenen Energie aus Fetten sollte 35 % der Gesamtenergie nicht überschreiten. Daraus berechnen sich bei einer täglichen Energiezufuhr von 2000 kcal ca. 60–80 g Gesamtfett pro Tag. Darüber hinaus ist es von besonderer Bedeutung, die Qualität des aufgenommenen Fettes zu beachten bzw. zu modifizieren. Einfach ungesättigte Fettsäuren sollen mindestens ein Drittel der gesamten Fettzufuhr ausmachen.

Maximal 10 % der täglichen Gesamtenergiezufuhr sollten, wie bei gesunden Erwachsenen, in Form von gesättigten Fettsäuren und Transfettsäuren aufgenommen werden.

Transfettsäuren entstehen bei der Hydrogenierung pflanzlicher Öle bzw. im Pansen von Wiederkäuern. Gesättigte Fettsäuren sind vor allem in tierischen Lebensmitteln und streichfähigen Fetten zu finden. Sie sind der diätetische Faktor mit den größtmöglichen Auswirkungen auf den Serumcholesterinspiegel. Eine tägliche Aufnahme von 5 g Transfettsäuren und mehr, erhöht das kardiovaskuläre Risiko um 25 % [28]. In verschiedenen Studien wurde ein LDL-Cholesterin-steigender und HDL-Cholesterin-senkender Effekt beobachtet. Die Frage, ob ein höherer Konsum von Transfettsäuren mit einem höheren Diabetesrisiko verbunden ist, kann derzeit nicht endgültig beantwortet werden. Die Minimierung der Aufnahme von Transfettsäuren erscheint jedenfalls angezeigt. In Europa ist ihr quantitativer Anteil in Margarinen aufgrund verbesserter Produktionsbedingungen jedoch vernachlässigbar. Zu berücksichtigen sind jedoch andere mögliche Quellen für Transfettsäuren wie Fastfood-Produkte und fettreiche Backwaren. Natürliche Transfette, die beispielsweise in Milchprodukten oder Rindfleisch enthalten sind, wurden in epidemiologischen Studien mit einem abgesenkten Diabetesrisiko in Zusammenhang gebracht [29].

Die Aufnahme mehrfach ungesättigter Fettsäuren (PUFA) sollten 10 % der täglichen Gesamtenergieaufnahme nicht überschreiten, wobei hier vor allem eine übermäßige Zufuhr von Omega-6-Fettsäuren vermieden werden soll.

Es gibt Hinweise, dass der Austausch von gesättigten Fettsäuren durch einfach oder mehrfach ungesättigte Fettsäuren einen protektiven Effekt in der Prävention der koronaren Herzkrankheit (KHK) hat. Der Austausch der gesättigten Fettsäuren mit Kohlenhydraten senkt das Risiko hingegen nicht. Eine Modifikation der Lebensmittelauswahl und der Ernährungsgewohnheiten kann auch eine deutliche Verbesserung im Sinne der sekundären Prävention der KHK bewirken [30]. Zur Klärung des Einflusses einer optimalen Fettsäurezusammensetzung auf die Diabetesprävention, Entwicklung des Serumcholesterinspiegels und des kardiovaskulären Risikos werden weitere Studien benötigt [31].

Fischölsupplemente können bei Menschen mit T2DM den Triglyzeridspiegel senken. Eine generelle Supplementierung mit Fischölen bei Personen mit Diabetes ohne ein kardiovaskuläres Risiko kann aber derzeit nicht empfohlen werden [32]. Nahrungsmittel, die reich an langkettigen Omega-3-Fettsäuren sind, wie beispielsweise fetter Fisch, Nüsse oder Samen, sind zur Prävention und in der Behandlung von kardiovaskulären Erkrankungen vorzuziehen [3]. Vegane „Fischöl“-Supplemente, die aus Algen gewonnen werden, sind ökologisch wesentlich besser.

### 2.4 Eiweiß

Der Anteil der täglichen Proteinaufnahme an der Gesamtenergiezufuhr kann bei Personen unter 65 Jahren und ohne Anzeichen einer Nephropathie 10–20 Energieprozent (E%), also 0,8–1,3 g pro kg Körpergewicht ([KG], Zielgewicht), betragen. Älteren und geriatrischen Menschen (> 65 Jahre) wird eine höhere Eiweißzufuhr von mindestens 1 g pro kgKG-Zielgewicht (15–20 E%) am Tag empfohlen, um eine Mangelernährung zu vermeiden [33–35]. Während einer energiereduzierten Diät zur Gewichtsabnahme ist darauf zu achten, dass die adäquate Proteinaufnahme sichergestellt ist.

Inwiefern eine höhere Proteinaufnahme (> 20 % der täglichen Energieaufnahme) sich langfristig auf die Entwicklung einer Nephropathie auswirkt, ist noch nicht endgültig geklärt. Die Proteinaufnahme in den üblichen Mengen (≈ 1 g pro kgKG) erscheint sicher [36, 37]. Der Blutglukosespiegel wird durch die Proteinaufnahme kaum erhöht, allerdings stimuliert Nahrungsprotein die Insulinsekretion [38]. Eine Beschränkung der Proteinaufnahme, um die Entwicklung einer Albuminurie und das Fortschreiten einer (diabetischen) Nephropathie zu reduzieren, wurde in der Vergangenheit empfohlen – die Ergebnisse vieler klinischer Studien, Metaanalysen und Reviews sind allerdings kontrovers [4, 34, 39]. In einer Metaanalyse konnte eine Proteinrestriktion auf 0,6–0,8 g pro kgKG keine Verbesserung der Nierenfunktion zeigen [40]. Extreme Restriktionen auf 0,3–0,4 g pro kgKG zeigten in einer Cochrane-Analyse eine gering signifikante Reduktion der Niereninsuffizienz [41, 42]. Solche derartigen Einschränkungen sind jedoch in der Praxis schwer durchführbar und bergen ein erhöhtes Risiko einer Mangelernährung, die wiederum mit einer erhöhten Mortalität im Zusammenhang steht [43]. Eine Proteinaufnahme gemäß den normalen Empfehlungen bei Personen mit diabetischer Nephropathie und einer glomerulären Filtrationsrate zwischen 60 ml/min und 45 ml/min konnte nicht mit einer rascheren Verschlechterung der Nierenfunktion in Zusammenhang gebracht werden [34]. Übereinstimmend mit dem Konsensuspapier der Amerikanischen Diabetesgesellschaft wird eine eingeschränkte Eiweißzufuhr bei Niereninsuffizienz nicht empfohlen [4]. In den letzten Jahren wurde der Einfluss einer proteinreichen, kohlenhydratarmen Diät auf das Ausmaß der Gewichtsabnahme sehr kontrovers diskutiert. Eine randomisierte Langzeitstudie untersuchte den Einfluss einer proteinreichen (30 E% Protein) vs. eine kohlenhydratreiche Ernährung (15 E% Protein) auf das Körpergewicht von Patient:innen mit T2DM. Die proteinreiche Ernährung hatte keinen signifikant besseren Einfluss auf das Körpergewicht und den Bauchumfang [36]. Es ist anzumerken, dass ein Proteinanteil von 30 % an der Gesamtenergieaufnahme nicht praktikabel zu sein scheint – im Mittel lag die Proteinaufnahme zwischen 20 und 21 E% [36]. Proteinreiche Diäten aus tierischen Quellen favorisieren in der Regel eine hohe Aufnahme von Cholesterin und gesättigten Fettsäuren – der Konsum von Obst und Gemüse wird stark eingeschränkt – sie müssen daher im Hinblick auf die Prävention einer Arteriosklerose kritisch betrachtet werden.

Es gibt zunehmend Hinweise, dass vermehrter Konsum an pflanzenbasiertem Protein und der Ersatz von tierischem Protein durch pflanzliches Protein mit einem geringeren Risiko für die Gesamtmortalität und kardiovaskuläre Mortalität, einer geringen Verbesserung des HbA_1c_ und der Nüchternglukose bei Erwachsenen mit Typ-2-Diabetes assoziiert ist [44–46]. Proteinreiche pflanzliche Quellen enthalten geringere Mengen an gesättigten Fettsäuren und mehr Ballaststoffe [47].

## 3. Mikronährstoffe

Die ausreichende Aufnahme von Mikronährstoffen (Vitaminen und Spurenelementen) ist ein wichtiger Faktor zur Erhaltung der Gesundheit von Menschen mit Typ-1- und Typ-2-Diabetes. Die empfohlene tägliche Zufuhr unterscheidet sich nicht von der für gesunde Erwachsene. Lebensmittel, die reich an Vitaminen und Spurenelementen sind, sollten daher bevorzugt werden.

Vor allem Schwangeren, Stillenden, Patient:innen nach metabolisch-bariatrischen Operationen, älteren Patient:innen und solchen, die eine energiereduzierte Ernährungsweise einhalten, kann eine Supplementierung mit einem Vitamin- bzw. Mineralstoffpräparat empfohlen werden. Eine ständige Supplementierung von Mikronährstoffen in Dosierungen über der empfohlenen Tagesmaximaldosis ist besonders beim Fehlen von klinischen bzw. laborchemischen Mangelzuständen abzulehnen.

Eine aktuelle Arbeit untersuchte die Auswirkungen von Informationen zu Nahrungsergänzungsmittel in den sozialen Medien. Diese sind für viele Personen eine bedeutende Informationsquelle. Soziale Medien können zwar eine wertvolle Plattform für evidenzbasierte Aufklärung bieten, gleichzeitig werden jedoch zahlreiche potenziell gesundheitsgefährdende Fehlinformationen und Desinformationen verbreitet, die teilweise zum Abbruch oder Veränderung wirksamer medizinischer Therapien zugunsten unwirksamer Nahrungsergänzungsmitteln führen können [48]. Daher ist die systematische Erfassung des Konsums von Nahrungsergänzungsmitteln sowie der hierzu genutzten Informationsquellen im Rahmen der Anamnese zunehmend zu berücksichtigen.

### 3.1 Nahrungsergänzungsmittel

#### 3.1.1 Vitamin B_12_

Eine Langzeit-Metformin-Gabe kann mit einem erniedrigten Vitamin‑B_12_-Spiegel assoziiert sein. Die Einnahme von Protonenpumpenhemmern kann die Bioverfügbarkeit von Vitamin B_12_ darüber hinaus reduzieren. Eine regelmäßige laborchemische Kontrolle und bei Bedarf eine Supplementierung von Vitamin B_12_ kann, insbesondere bei Patient:innen mit Anämie oder peripherer Neuropathie, nach Magenresektion, Typ-A-Gastritis sinnvoll sein [3, 49].

#### 3.1.2 Zink

Zink ist als Co-Faktor der Superoxiddismutase im Radikalstoffwechsel von Bedeutung. Eine Supplementierung kann Störungen der Wundheilung positiv beeinflussen [50]. Die Evidenz für eine Supplementierung bei Menschen mit Diabetes ist als unzureichend anzusehen [51, 52].

#### 3.1.3 Vitamin D

Ein Vitamin-D-Mangel steht im Zusammenhang mit Veränderungen des Glukosemetabolismus und der Insulinsekretion. Ein Umbrella-Review zeigte, dass niedrige Serum-Vitamin-D-Spiegel die Insulinsensitivität verschlechtern können und eine Supplementierung vor allem bei nachweisbarem Vitamin-D-Mangel zu moderaten Verbesserungen von HbA_1c_, Nüchternblutzucker und Insulinresistenz führen kann [53, 54]. Jedoch ist die Evidenz für eine generelle Vitamin-D-Supplementierung basierend auf systematischen Reviews und Metaanalysen als widersprüchlich anzusehen [53, 55–57].

#### 3.1.4 Chrom

Eine unzureichende Chromzufuhr wird mit einer gestörten Glukosetoleranz in Verbindung gebracht. Der Nachweis einer blutzuckersenkenden Wirkung ist derzeit nicht ausreichend durch wissenschaftliche Evidenz gestützt [58, 59]. Für die Bestimmung einer optimalen Dosierung sowie evidenzbasierten Nachweis eines gesundheitlichen Nutzens sind weitere Studien erforderlich.

#### 3.1.5 Selen

Der Selenstatus wird im Zusammenhang mit einem Diabetesrisiko und einem möglichen positiven Einfluss auf die glykämische Kontrolle von Personen mit Diabetes diskutiert. Sowohl ein niedriger als auch ein hoher Selenplasmaspiegel scheinen sich ungünstig auszuwirken (U-förmiger Zusammenhang). In einem systematischen Review wird festgehalten, dass die Evidenz für eine routinemäßige Supplementierung von Selen unzureichend ist [60].

#### 3.1.6 Kalzium und Magnesium

Bei älteren Patient:innen mit T2DM, vor allem mit niedrigem BMI, wurde eine höhere Prävalenz für Osteoporose gefunden [61]. Eine optimale Kalziumresorption ist nur bei gleichzeitig verfügbarem Vitamin D erreichbar. Es gibt Hinweise, dass eine Supplementierung mit Kalzium und Vitamin D mit einem geringeren Risiko eines T2DM verbunden ist. Allerdings muss die Evidenz dafür noch als unzureichend angesehen werden.

Ein unzureichender Magnesiumstatus wird mit einer schlechten glykämischen Kontrolle bei gestörter Glukosetoleranz [62] und mit T2DM und assoziierten Komorbiditäten [63] in Zusammenhang gebracht. Die ausreichende Magnesiumaufnahme scheint die Progression einer eingeschränkten Glukosetoleranz zu T2DM zu verzögern [64, 65]. Die Evidenz für eine Magnesiumsupplementierung bei Menschen mit T2DM ist allerdings unzureichend sowie widersprüchlich.

#### 3.1.7 Alpha-Liponsäure

Alpha-Liponsäure wird als Supplement oder Arzneimittel zur Behandlung diabetischer Polyneuropathie eingesetzt. Aktuelle Reviews zeigen jedoch im Vergleich zu Placebo nur geringe bis keine Wirkung auf neuropathische Symptome. Insgesamt ist die Evidenzlage für einen klinischen relevanten Nutzen begrenzt, und somit kann eine routinemäßige Supplementierung nicht empfohlen werden [66, 67].

#### 3.1.8 Antioxidanzien

Da Diabetes mit erhöhtem oxidativem Stress verbunden ist, erscheint es möglich, dass bei schlecht kontrolliertem diabetischem Stoffwechsel der Bedarf an Antioxidanzien erhöht ist. In verschiedenen Studien wurde eine inverse Beziehung zwischen der Antioxidanzienzufuhr und dem KHK-Risiko gefunden [68, 69]. Die deutlichste Beziehung bestand für Tocopherole und β‑Karotin, der Effekt der Ascorbinsäure war geringer ausgeprägt. Klinische Studien, die den Effekt einer Tocopherol-Supplementierung in der Sekundärprävention der KHK untersuchten, kamen zu widersprüchlichen Ergebnissen. Die Supplementierung mit β‑Karotin zeigte keinen positiven Effekt, bei Raucher:innen wurde sogar ein höheres Krebsrisiko attestiert. Eine Supplementierung mit Antioxidanzien kann derzeit aufgrund ungeklärter Effektivität und unbekannter Langzeitfolgen nicht empfohlen werden [69].

#### 3.1.9 Pflanzliche Nahrungsergänzungsmittel

Die Verwendung von komplementären und alternativmedizinischen Produkten ist bei Patient:innen mit Diabetes weit verbreitet. Zimt zählt dabei zu den am häufigsten verwendeten pflanzlichen Nahrungsergänzungen [70]. Zimt werden antioxidative, antiinflammatorische und antibakterielle Eigenschaften zugeschrieben. Bisher sind mehr als 200 Zimtarten bekannt [71]. Diese unterscheiden sich in der Zusammensetzung ihrer Inhaltsstoffe zum Teil signifikant. Dies und die Abhängigkeit des Gehalts der Inhaltsstoffe von Klima, Wetter, Bodenbeschaffenheit und Variationen in der Herstellung machen eine Standardisierung (d. h. immer gleicher Gehalt der Wirksubstanz) von Zimt schwierig. Die Verwendung größerer Mengen von Zimt, welcher zum Würzen beim Kochen und Backen verwendet wird, kann daher nicht zielführend sein.

Die Wirkung von Zimt auf die glykämische Kontrolle wurde bisher in unterschiedlicher Dosierung an kleinen Studienpopulationen mit unterschiedlicher Dauer (40 Tage bis 4 Monate) untersucht. Die Ergebnisse sind entsprechend inhomogen. Die Evidenz für eine Empfehlung der Supplementierung mit Zimt reicht nicht aus [71, 72].

Die im Tierversuch gefundenen toxischen Effekte auf die Nierenfunktion werden kontrovers diskutiert [71]. Cassia-Zimt enthält darüber hinaus Cumarin, weshalb sich eine länger dauernde Einnahme auf die Blutgerinnung auswirken kann. Ceylon-Zimt hingegen enthält geringere Mengen Cumarin [71].

## 4. Lebensmittelbezogene Empfehlungen

Neben makro- und mikronährstoffbezogenen Empfehlungen stellen lebensmittelbezogene Empfehlungen eine praxisnahe Ergänzung dar, da diese den Patient:innen eine konkrete Umsetzung im Alltag ermöglichen.

### 4.1 Getreideprodukte/Vollkorn

Eine Ernährung reich an wenig verarbeiteten Vollkornprodukten, Hülsenfrüchten, Salat und Gemüse kann eine normo- und hypokalorische Energieaufnahme unterstützen und zu einer Gewichtsreduktion beitragen. Durch den reichlichen Verzehr von Lebensmitteln mit einer hohen Nährstoffdichte, also Lebensmittel, die einen hohen Gehalt an Nährstoffen in Relation zu ihrem Energiegehalt haben, wird eine gesteigerte Zufuhr gesundheitsfördernder Inhaltsstoffe wie Ballaststoffe, Mikronährstoffe und sekundäre Pflanzenstoffe bei gleichzeitig geringer Zufuhr stark verarbeiteter Nahrungsmittel mit gesundheitlich nachteiligen Inhaltsstoffen erreicht. Eine hohe Ballaststoffzufuhr wirkt sich positiv auf die Sättigung, auf die Verdauung und Darmgesundheit sowie den Cholesterinspiegel aus und reduziert das Risiko für kardiovaskuläre Erkrankungen [13, 73–75].

In der Ernährungstherapie des Diabetes ist der Verarbeitungsgrad von Vollkornprodukten von Bedeutung. Jenkins et al. belegten bereits 1988 unterschiedliche postprandiale Auswirkungen von Vollkornbroten mit verschiedenem Gehalt an Vollkornmehl und grobkörnigem Getreide. Ein hoher Anteil an ganzen Getreidekörnern führt zu geringeren Blutglukosewerten. Die Samen- und Fruchtschalen des Getreides stellen für die Amylase eine physikalische Barriere dar und verlangsamen den Verdauungs- und Stoffwechselprozess. Vollkornprodukte mit einem hohen Anteil an ganzen Körnern bewirken einen geringen postprandialen Glukoseanstieg und sind für Menschen mit Diabetes ein wesentlicher, nichtmedikamentöser Therapiebestandteil [76].

Beim Konsum kohlenhydratbetonter Nahrungsmittel sollen Menschen mit Diabetes die tatsächliche Verzehrmenge und den Ballaststoffgehalt der Nahrungsmittel beachten, um die glykämische Reaktion autonom und positiv zu beeinflussen. Stark verarbeitete Vollkornprodukte und Getreideerzeugnisse aus fein vermahlenem Vollkorn sind für einen stabilen Glukoseverlauf begrenzt geeignet, da sie einen raschen postprandialen Blutglukoseanstieg bewirken können. Hierbei ist die Kombination dieser Nahrungsmittel mit weiteren Mahlzeitenkomponenten wie Proteinen und Fetten bzw. Ballaststoffen ausschlaggebend.

### 4.2 Gemüse und Obst

Eine Ernährung, die vor allem reich an Gemüse ist, kann eine Gewichtsreduktion sowie die Versorgung mit Mikronährstoffen unterstützen. Während es bei Gemüse und Salat keine einschränkenden Verzehrempfehlungen gibt, können sich größere Mengen Obst, besonders der Konsum von Fruchtsäften oder Smoothies negativ auf die Blutzuckerkontrolle auswirken.

Die Weltgesundheitsorganisation (WHO) empfiehlt, zur Prävention von T2DM täglich mindestens 400 g bzw. 5 Portionen Gemüse und/oder Obst, vorzugsweise aus frischem regionalem und saisonalem Angebot, zu verzehren [77]. Als empfehlenswerte Mengen haben sich in der Praxis maximal 2 Handvoll Obst pro Tag und mindestens 3 große Handvoll Gemüse und Salat pro Tag erwiesen [47].

Die empfohlenen Mengen Obst stellen eine wichtige Quelle für die Zufuhr an Vitaminen, Mineralstoffen, Spurenelementen und Ballaststoffen dar und machen Obst zu einer relevanten Lebensmittelgruppe in der ausgewogenen Ernährung von Menschen mit Diabetes. Durch gezielte Kombinationen von Obst mit beispielsweise proteinreichen Nahrungsmitteln wie einem naturbelassenen gesäuertem Milchprodukt kann der Blutglukoseverlauf günstig beeinflusst werden [78–80].

Patient:innen mit prandialer Insulintherapie sollen beim Verzehr von Obst die entsprechenden KE berücksichtigen und die Insulinapplikation anpassen. Der Kohlenhydratgehalt von stärkereichen Gemüsesorten, wie z. B. Kartoffeln, Süßkartoffeln oder größere Mengen Kürbis, muss ebenfalls mit Insulin abgedeckt werden.

Energiereiche und nährstoffarme Lebensmittel durch die empfohlene Menge an frischem Obst zu ersetzen kann eine Gewichtsreduktion unterstützen [76, 81]. Der Konsum großer Mengen an Früchten und zuckerreichen Obsterzeugnissen wie Fruchtsäfte, Smoothies, zuckerreichen Convenience-Produkten und Trockenfrüchten soll vermieden werden, um unerwünschte Blutglukosereaktionen zu verhindern (klinische Erfahrung). Darüber hinaus unterscheiden sich ganze Früchte hinsichtlich ihres Ballaststoffgehalts von Fruchtsäften und tragen somit zu einer besseren Sättigung bei [82].

### 4.3 Hülsenfrüchte

Hülsenfrüchte zählen zu den bedeutendsten pflanzlichen Proteinquellen [82]. Hülsenfrüchte sollten vermehrt als Fleischersatz gegessen werden und wirken sich durch ihren Anteil an löslichen Ballaststoffen und resistenter Stärke positiv auf Blutzucker‑, Lipid- und Insulinspiegel aus [83, 84]. Der Konsum von 75 g Leguminosen pro Tag wirkt sich aus ökologischen und sozialen Gründen laut Empfehlungen der EAT Lancet Commission positiv auf die Gesundheit aus [47, 85]. Die Blutzuckerwirksamkeit von Hülsenfrüchten muss individuell ausgetestet werden, und eine Berücksichtigung größerer Mengen im Rahmen einer prandialen Insulintherapie kann notwendig sein [83, 84]. In die entsprechende Lebensmittelgruppe werden neben Hülsenfrüchten auch weitere pflanzliche Proteinquellen wie Tofu, Tempeh und texturiertes Soja- oder Erbsenprotein (z. B. Sojaschnetzel oder -granulat) einbezogen [82]. Stark verarbeitete pflanzliche Fleischersatzprodukte enthalten meistens einen hohen Anteil an Fett, gesättigten Fettsäuren, Salz, Zusatzstoffen oder Zucker und sollten nur selten verzehrt werden [86].

### 4.4 Milchprodukte

Milch und Milchprodukte sind aufgrund ihrer Nährstoffdichte und ihres hohen Proteingehalts ein wichtiger Bestandteil der gesunden Ernährung bei Diabetes. Es ist dabei nicht notwendig, auf fettarme Milchprodukte zurückzugreifen [87].

Die Datenlage zeigt positive Effekte von Milchfett bei T2DM sowie eine kardioprotektive Wirkung [88]. In Interventionsstudien wurde festgestellt, dass ein vermehrter Verzehr von Milchprodukten die Insulinresistenz verbessert. Fermentierte Milchprodukte wie Joghurt können das Risiko für T2DM ebenfalls senken [87]. Zudem wird eine positive Auswirkung von Milchsäurebakterien auf das Mikrobiom vermutet [88, 89]. Aktuelle Studien zeigen einen protektiven Effekt von vollfetten Milchprodukten auf das metabolische Syndrom auf [90]. Es konnte kein Zusammenhang zwischen gesättigten Fettsäuren aus Milch und einem höheren Risiko für KHK nachgewiesen werden [91].

Käse, als Vorspeise gegessen, erhöht die Magenverweildauer der Speisen und führt zu einem geringeren Blutzuckeranstieg [79]. Trotz des Gehalts an gesättigten Fettsäuren und der Kalorien konnte bisher kein Nachteil von Käsekonsum auf das Diabetesrisiko festgestellt werden [87, 92].

Aktuelle Studien zeigen keinen Zusammenhang zwischen Butterkonsum und KHK, jedoch eine protektive Wirkung auf Diabetes [93]. Butter sollte aufgrund des Fettgehalts trotzdem nur in Maßen gegessen werden.

Molkenprotein hat blutzuckersenkende Effekte und trägt zu einer lang anhaltenden Sättigung bei. Zusätzlich stimuliert es die Muskelproteinsynthese stärker als andere Proteinquellen [94].

Da Milchzucker eine kaum blutzuckererhöhende Wirkung aufweist, sollten Milchprodukte ohne Zuckerzusatz bevorzugt werden [89]. Handelsübliche Fruchtjoghurts sind meist zuckerreich, alternativ empfiehlt sich stattdessen Naturjoghurt mit etwas frischem Obst oder einem kleinen Löffel Marmelade.

In den letzten Jahren ist ein kontinuierlicher Anstieg des Interesses an „Pflanzendrinks“ zu verzeichnen. Dazu zählen z. B. Soja‑, Mandel‑, Hafer‑, Kokos- und Reisdrinks. Da pflanzliche Getränke (ausgenommen Sojadrink) im Vergleich zu Kuhmilchprodukten abweichende Nährstoffprofile aufweisen, können sie ernährungsphysiologisch nicht als gleichwertiger Ersatz eingestuft werden [82]. Jene Pflanzendrinks auf Soja- oder Erbsenbasis weisen einen Proteingehalt auf, der dem von Kuhmilch entspricht. Pflanzendrinks sind laktosefrei, weisen einen höheren Anteil an ungesättigten Fettsäuren auf und werden teilweise v. a. mit Calcium und Vitamin B_12_ angereichert, um den geringeren Gehalt an Vitaminen und Mineralstoffen auszugleichen [82, 95]. Die Zusammensetzung der Drinks unterscheiden sich je nach Marke bzw. von Bio-Qualität (diese sind meistens nicht angereichert). Daher sollte ihre Nährstoffzusammensetzung in jedem Fall mithilfe der Nährstoffkennzeichnungstabelle genauer betrachtet werden. Es sind Varianten ohne Zuckerzusatz zu bevorzugen. Jene Alternativen auf Getreidebasis (z. B. Reis- oder Haferdrink) weisen außerdem einen deutlich höheren Kohlenhydratgehalt auf und sind somit kein geeigneter Ersatz [95].

### 4.5 Fleisch

Mageres Fleisch ist eine gute Proteinquelle und liefert Vitamine sowie Mineralstoffe. Verarbeitete Fleisch- und Wurstwaren sollten aufgrund ihres häufig hohen Gehalts an Fett, Natrium, Nitrit, Zusatzstoffen und Hämeisen möglichst vermieden werden [96]. Rotes Fleisch und Wurstwaren durch andere Proteinquellen zu ersetzen, wirkt sich günstig auf Entzündungsprozesse und den Glukosestoffwechsel aus und senkt das Risiko für Krebs [97]. In Beobachtungsstudien steht eine fleischbetonte Ernährung in Verbindung mit einer erhöhten kardiovaskulären Mortalität [98]. Zudem besteht vor allem bei verarbeitetem rotem Fleisch eine moderate Beziehung mit Krebserkrankungen, KHK sowie T2DM [99–101]. Der Ersatz durch alternative Proteinquellen wie Hülsenfrüchte, Fisch oder Nüsse ist dagegen mit einem geringeren Erkrankungsrisiko assoziiert [100]. Eine hohe Aufnahme von Hämeisen steigert das Risiko für KHK und begünstigt eine Insulinresistenz. Hämeisen erschwert zudem die Glukoseaufnahme in den Muskel. Leguminosen, Hühnerfleisch, Eier, Fisch, Milchprodukte, Nüsse und Vollkorngetreide sollten als Eiweißquelle bevorzugt werden [102, 103].

### 4.6 Fisch

Für Menschen mit T2DM können die allgemeingültigen Empfehlungen der Österreichischen und Deutschen Gesellschaft für Ernährung, 1 bis 2 Portionen Fisch pro Woche zu verzehren, herangezogen werden.

Der regelmäßige Verzehr von Fisch, insbesondere von fettreichem Fisch (z. B. Lachs, Hering, Makrele), kann das Lipoproteinprofil im Blut positiv beeinflussen und das Risiko für die KHK-Mortalität und den ischämischen Schlaganfall herabsetzen [104]. Die positive Wirkung wird laut wissenschaftlicher Datenlage v. a. durch die Aufnahme der langkettigen Omega-3-Fettsäuren Eicosapentaensäure (EPA) und Docosahexaensäure (DHA) erreicht, wobei hier eine Aufnahme von 250 mg EPA und DHA pro Tag genügt, um den durch koronare Herzkrankheit bedingten Todesfällen vorzubeugen [105]. Diese Menge lässt sich durch den Verzehr von ein bis wahlweise 2 Fischmahlzeiten pro Woche, wenn davon 70 g fettreicher Fisch verzehrt werden, abdecken. Allerdings variieren die Gehalte an EPA und DHA je nach Fischart, Fanggebiet, Nahrung oder Fütterung und Zubereitungsart, weshalb die Verzehrmenge von 70 g nur als Orientierungswert angesehen werden kann [106–108]. Eine ausreichende Evidenz, zur Empfehlung eines höheren Fischverzehrs (> 1 bis 2 Portionen/Woche) bei Menschen mit T2DM sowie zur Supplementation langkettiger Omega-3-Fettsäuren (DHA und EPA) liegt nicht vor [32, 109]. Ebenso ist ein deutlich höherer Fischverzehr aus Gründen der Nachhaltigkeit und des Risikos einer zu hohen Aufnahme an unerwünschten Schadstoffen insbesondere bei langlebigen Seefischen (z. B. Methylquecksilber) nicht empfehlenswert. Dennoch herrscht nach Angaben wissenschaftlicher Literatur Einigkeit darüber, dass bei einem Fischverzehr von 1 bis 2 Portionen pro Woche der gesundheitliche Nutzen durch Fischverzehr überwiegt [105, 110]. Beim Kauf von Fisch sollte auf eine nachhaltige Herkunft geachtet werden. Fisch aus Wildfang bietet gegenüber solchem aus Aquakulturen einen ernährungsphysiologischen Vorteil, da eine bessere Fettsäurezusammensetzung vorliegt [111]. Für den Verzehr von Fisch an sich kann aus Sicht der Datenlage keine signifikante Assoziation hinsichtlich des Risikos für T2DM festgestellt werden [112]. Im Gegensatz dazu werden gesamte Ernährungsmuster, die Fisch einschließen (z. B. mediterrane Ernährung), mit einem geringeren Risiko für Diabetes in Verbindung gebracht [113].

Abgesehen von den enthaltenen Omega-3-Fettsäruen EPA und DHA gibt es zahlreiche weitere Vorteile von Fischverzehr. Fisch ist ein nährstoffreiches Lebensmittel, das u. a. eine gute Quelle für Vitamin D, Jod, Selen und hochwertiges Protein darstellt [104].

### 4.7 Eier

Eier liefern hochwertiges Protein, zahlreiche essenzielle Nährstoffe, darunter fettlösliche Vitamine, B‑Vitamine sowie Mineralstoffe und Cholin [114–116]. Ein Ei enthält durchschnittlich etwa 240 mg Cholesterin, daher wurde lange Zeit die Zufuhr kontrovers diskutiert [116, 117]. Der Eiverzehr steht jedoch in einem deutlich geringeren Zusammenhang mit einem erhöhten Serumcholesterinspiegel als die Aufnahme gesättigter Fettsäuren und Transfettsäuren. Aktuelle Studien deuten darauf hin, dass ein moderater Eierkonsum nicht mit einem erhöhten Risiko für kardiovaskuläre Ereignisse oder T2DM assoziiert ist. Die Gesamtevidenz legt nahe, dass die Qualität des gesamten Ernährungsverhaltens entscheidender ist als der isolierte Eierkonsum [115–118].

### 4.8 Öle, Nüsse und Samen

Tierische und pflanzliche Fette unterscheiden sich aufgrund ihres Fettsäuremusters in ihren gesundheitlichen Auswirkungen. Die Mehrzahl der ungesättigten pflanzlichen Öle (ausgenommen Palm- und Kokosnussöl) weisen positive Effekte auf den Fettstoffwechsel und koronare Herzerkrankungen auf (insbesondere der positive Einfluss von Olivenöl wird in zahlreichen Forschungsarbeiten hervorgehoben) [119–121]. Hingegen sind gesättigte Fette, mit Ausnahme von Milchfett, mit nachteiligen Wirkungen assoziiert [82]. Besonders Pflanzenöle mit einem hohen Gehalt an Omega-3-Fettsäuren, wie Leinöl, Walnussöl, Hanföl oder Rapsöl, sowie Nüsse und Samen stellen wertvolle Quellen gesundheitsförderlicher Fettsäuren dar. Sonnenblumen‑, Distel- oder Maiskeimöl sind hingegen wegen des höheren Gehaltes an Omega-6-Fettsäuren weniger empfehlenswert.

### 4.9 Getränkeauswahl

Um Blutglukosespitzen zu vermeiden, sollten Menschen mit Diabetes motiviert werden, ausschließlich kohlenhydratfreie Getränke wie Wasser, Mineralwasser und ungesüßten Tee zu sich zu nehmen, da dies zu einer ausgewogeneren Ernährung beiträgt und die Kaloriendichte reduziert werden kann. Fruchtsaftgetränke und Softdrinks sind aufgrund ihres hohen Zuckergehalts nur zur Behandlung einer Hypoglykämie geeignet. Süßstoffhaltige Light-Limonaden oder Sirupe, können, wenn unbedingt nötig, in Maßen konsumiert werden. Durch den süßen Geschmack reduziert sich jedoch bei vermehrtem Konsum die individuelle Süßschwelle und führt häufig zu größerem Verlangen nach süßen Getränken und Lebensmitteln. Eine randomisierte kontrollierte Studie bei Frauen mit T2DM zeigte, dass der Ersatz von Diätgetränken durch Wasser zu einem stärkeren und längerfristigen Gewichtsverlust sowie zu einer signifikant höheren Remissionsrate von T2DM führte im Vergleich zum Weiterkonsum von Light-Getränken. Eine Umstellung auf Wasser kann die Vorliebe für Süßes reduzieren, sodass süße Lebensmittel seltener konsumiert werden. Außerdem kann die eingesparte Kalorienmenge durch Light-Getränke auch überschätzt werden und zu einer höheren Energieaufnahme aus anderen Lebensmitteln führen [122].

#### 4.9.1 Alkoholkonsum

Menschen mit Diabetes sollten über die Risiken und Auswirkungen von Alkoholkonsum auf den Blutzuckerspiegel sowie auf eine mögliche Verschlechterung der Stoffwechseleinstellung aufgeklärt werden. Durch die Beeinträchtigung der Gegenregulationsmechanismen geht mit dem Genuss von Alkohol ein erhöhtes Risiko für v. a. nächtliche Hypoglykämien einher. Besonders zuckerreiche alkoholische Getränke wie Bier oder Mischgetränke können außerdem zu einem erhöhten Blutglukosespiegel führen. Zudem darf der Energiegehalt von Alkohol mit 7 kcal/g nicht außer Acht gelassen werden. Nach Einschätzung der WHO gibt es keine Menge an Alkoholkonsum, die als gesundheitlich unbedenklich eingestuft werden kann [123]. Im Positionspaper der Deutschen Gesellschaft für Ernährung wird das Risiko für negative Gesundheitsfolgen in Abhängigkeit vom Alkoholkonsum pro Woche in risikoarm, moderat sowie riskant unterteilt. Ein risikoarmer Alkoholkonsum entspricht 1 bis 2 alkoholischen Getränken pro Woche [124]. Weitere Details s. Leitlinie „Rauchen, erhitzte Tabakprodukte, Alkohol und Diabetes mellitus“.

### 4.10 Zucker und Süßungsmittel

Die WHO empfiehlt weniger als 10 % der Gesamtenergiezufuhr aus zugesetztem Zucker zuzuführen. Eine weitere Reduzierung auf 5 % der täglichen Energiezufuhr könnte laut WHO zusätzliche gesundheitliche Vorteile bieten, insbesondere in Bezug auf die Prävention von Übergewicht. Für Menschen mit Diabetes gibt es keine eigenen Empfehlungen. Da Saccharose in isolierter Form, z. B. in Getränken, einen starken Blutglukoseanstieg verursacht, sollten Patient:innen motiviert werden, Haushaltszucker weitgehend zu vermeiden und, wenn nötig, durch Alternativen zu ersetzen, die keinen Einfluss auf den Blutglukoseverlauf haben [125, 126]. Eine Reduktion der Aufnahme von Mono- und Disacchariden (Glukose, Fruktose, Glukose‑, Fruktosesirup, Saccharose) in verarbeiteten Lebensmitteln und „Getränken“ erleichtert das Erreichen einer ausgeglichenen bzw. negativen Energiebilanz und damit die Gewichtsstabilisierung bzw. eine Gewichtsreduktion sowie eine Reduktion des Risikos für kardiovaskuläre Erkrankungen und die Entstehung einer Fettleber [4, 127]. Alternative Süßungsmittel, wie beispielsweise Honig, Agavendicksaft, Ahornsirup, Kokosblütenzucker oder Dattelsirup, stellen aufgrund des ähnlichen Energiehaushalts und ebenfalls hoher Blutzuckerwirksamkeit keinen geeigneten Ersatz für Haushaltszucker dar.

Eine Ernährungsweise mit einem hohen Anteil an Haushaltszucker (> 20 % der Gesamttagesenergie) führt sowohl bei Menschen ohne Diabetes als auch bei Personen mit metabolischem Syndrom zu erhöhten Plasmatriglyzeriden [128]. Die Reaktion der Triglyzeride auf Nahrungszucker ist abhängig von der aufgenommenen Menge und dem gleichzeitigen Konsum anderer Lebensmittel. Dem Zuckerkonsum von Patient:innen mit metabolischem Syndrom (hohe Plasmaglukose‑, Triglyzerid-, niedrige HDL-Cholesterinspiegel) muss besondere Aufmerksamkeit gewidmet werden.

Süßstoffe können das Erreichen einer negativen Energiebilanz unterstützen [129]. Nach derzeitigem Wissen sind sie unter Einhaltung des ADI(„acceptable daily intake“)-Werts unbedenklich. Ein möglicher negativer Einfluss auf das Mikrobiom und die Glukosetoleranz wird diskutiert [130]. Ein zeitweiser moderater Konsum von Süßstoffen dürfte nach derzeitigem Stand keinen negativen Einfluss auf die Glukose- und Insulinregulierung bei T2DM haben [131–133]. Zuckeralkohole (Erythritol, Xylitol, Sorbit, Maltitol) werden in industriell hergestellten Lebensmitteln als Zuckeraustauschstoff verwendet, da sie weniger Energie enthalten und einen deutlich geringeren postprandialen Glukoseanstieg als Saccharose verursachen [134, 135]. In hohen Mengen können Zuckeralkohole abführend wirken. Von der Europäischen Behörde für Lebensmittelsicherheit (EFSA) werden sie als „generally recognized as safe“ zugelassen [134]. Witkowski et al. stellte in 2 Arbeiten einen möglichen Zusammenhang von Erythrit und Xylit mit einem erhöhten kardiovaskulären Risiko und gesteigerter Plättchenreaktivität her [136, 137]. Für endgültige Aussagen über Süßstoffe sowie Zuckeralkohole bedarf es jedoch weiterer Forschung und Langzeitstudien. Es ist jedenfalls darauf zu achten, dass die kalorische Einsparung durch die Verwendung von nichtkalorischen Süßstoffen und Süßungsmitteln nicht über andere Lebensmittel oder Getränke kompensiert wird.

## 5. Mahlzeitenfrequenz

Mehr als 3 Mahlzeiten pro Tag können oftmals einen Mitgrund einer Gewichtszunahme darstellen. Dies scheint durch eine insgesamt erhöhte Energieaufnahme und den durch häufige Mahlzeiten verursachten erhöhten Insulinspiegel verursacht zu werden [138, 139]. Zudem wird durch häufige Zwischenmahlzeiten das natürliche Hungersignal unterdrückt.

Während frühere Diabetestherapien aufgrund des hohen Hypoglykämierisikos Zwischenmahlzeiten oft notwendig machten, ist es mit den heutigen modernen Therapiemöglichkeiten meist nicht notwendig, Zwischenmahlzeiten zu essen. Die Entscheidung, welche Mahlzeitenfrequenz für welchen Menschen mit Diabetes optimal ist, sollte individuell, angepasst an die persönlichen Bedürfnisse und die Diabetestherapie, getroffen werden. Zur Gewichtsreduktion bzw. -stabilisierung und für gleichmäßige Blutglukoseverläufe empfiehlt es sich meist, ein 3‑Mahlzeiten-Prinzip einzuhalten. Intermittierendes Fasten wird im Abschn. 6.3 näher diskutiert.

Es wird häufig das Konzept diskutiert, Gemüse und Eiweiß vor den Kohlenhydraten zu essen, um Blutzuckerspitzen zu vermeiden. Ein Cross-Over-Studie zeigte, dass ein Molkenprotein-Shot (15 g) vor den Mahlzeiten Hyperglykämien senken und die euglykämische Zeit verlängern kann, allerdings handelt es sich hier um kleine Probandenzahlen und kurzfristige Untersuchungen. Es sind weitere Studien erforderlich [140]. Einige systematische Reviews und Interventionsstudien mit jedoch geringeren Proband:innenzahlen zeigen, dass die Reihenfolge (Gemüse > Eiweiß > Kohlenhydrate) kurzfristig zu geringeren postprandialen Glukose- und Insulinspitzen führen kann [141–145]. Die bisherige Evidenzlage ist jedoch begrenzt. Die Studien sind meist kurzfristig, mit kleinen Stichproben und heterogenen Designs; Langzeitdaten (z. B. zu Änderungen des HbA_1c_) und klinische Endpunkte fehlen, und Daten bei manifestem T2DM sind limitiert. Die beobachteten Effekte sind möglicherweise weniger auf die Reihenfolge der Nahrungsaufnahme zurückzuführen, sondern vielmehr auf die erhöhte Aufmerksamkeit für ballaststoffreiches Gemüse und Eiweiß als Mahlzeitenbestandteile. Dieses Konzept kann für manche Personen eine leicht verständliche Hilfestellung darstellen, um die Aufnahme ballastoff- und eiweißreicher Lebensmittel zu fördern. Wichtig ist jedoch, individuell abzuwägen, ob die Empfehlung einer Essensreihenfolge eine praktikable Unterstützung oder eher eine zusätzliche Belastung darstellt, zudem sie im Alltag nicht immer umsetzbar ist. Zur Bewertung der klinischen Relevanz sind größere und längerfristige Studien erforderlich.

## 6. Ernährungsformen und Gewichtsreduktion

Da 60–90 % der Personen mit T2DM auch von Übergewicht/Adipositas betroffen sind, stellt eine Gewichtsreduktion eine außerordentlich wichtige Säule in der Diabetestherapie dar.

### 6.1 Low-Carbohydrate/Low-fat

Eine generelle Reduktion der Kohlenhydrataufnahme zur Verbesserung der Stoffwechsellage wird immer wieder diskutiert. Diese Reduktion wird üblicherweise dann unter dem Terminus „Low-Carb-Diät“ subsummiert. Der Ausdruck „low-carb“ ist eigentlich falsch – es müsste „low-carb, high-fat“(LCHF)-Diät genannt werden [146]. Nach derzeitiger Definition spricht man von einer LCHF-Diät, wenn 50–150 g Kohlenhydrate pro Tag verzehrt werden. Eine Kohlenhydratreduktion wird je nach Intensität in „very-low-carb“, „low-carb“ und „moderate-carb“ klassifiziert. Letzteres wird mit 130–230 g (26 E%–45 E%) definiert [147]. Eine ketogene Diät, die Extremform der LCHF-Diät, erlaubt einen Kohlenhydratverzehr von 20–50 g pro Tag [148, 149]. Ziel der LCHF-Ernährung bzw. ihrer Extremform, der ketogenen Ernährung, ist, dass durch die Kohlenhydratreduktion weniger Glukose als Energielieferant zur Verfügung steht, der Insulinspiegel sinkt und der Körper durch Lipolyse Energie gewinnt. Nach dieser Hypothese müsste es einen Wert geben, ab dem diese metabolischen Veränderungen auftreten. Eine Kohlenhydratreduktion auf unter 45 % der aufgenommenen Energie kann zu Therapiebeginn mit einer stärkeren Reduktion des HbA_1c_ assoziiert sein. Langfristig ist sie einer Diät mit einem höheren Kohlenhydratanteil nicht überlegen [6, 150].

Zum derzeitigen Zeitpunkt fehlen gute Vergleichsstudien, ob eine LCHF-Ernährung einer „Low-fat, high-carb“-Ernährung bei Menschen mit Diabetes wirklich zu bevorzugen ist. Eine Metaanalyse zeigte, dass zumindest über einen kurzen Zeitraum eine LCHF-Diät zu einer Verbesserung der Blutzuckereinstellung und zu einer Gewichtsabnahme bei Personen mit T2DM führt [151]. Eine Metaanalyse zeigte, dass „low-carb“ (< 130 g KH/Tag) gegenüber „low-fat“ nach 6 Monaten zu einer höheren Diabetesremission führen könnte, indem signifikant mehr Patienten einen HbA_1c_ unter 6,5 % erreichten. Allerdings waren die Unterschiede bei Remission < 6,5 % ohne Medikation und längerer Intervention nicht mehr signifikant. Die Verbesserung von Triglyzeriden und Insulinsensitivität sowie Gewichtsverlust waren vor allem nach 6 Monaten zu beobachten, die sich jedoch nach 12 Monaten verringerten [152, 153]. Zu ähnlichen Ergebnissen kommt auch eine neuere Metaanalyse. Langfristige Daten fehlen jedoch [154]. Eine „Very-low-carb“-Diät (< 10 E%) erreichte nach 6 Monaten eine weniger wirksame Gewichtsreduktion als eine Low-carb-Diät. Das lässt sich wiederum durch die mangelnde Adhärenz dieser Ernährungsform erklären. Nach 6 Monaten erreichten die Patient:innen keinen signifikanten Unterschied in der Lebensqualität, jedoch nach 12 Monaten eine klinisch bedeutsame, aber statistisch nicht signifikante Verschlechterung der Lebensqualität [152, 155].

Noch deutlicher sind diese Resultate bei einer ketogenen Diät sichtbar. Allerdings, wie bei allen extremen Ernährungsformen, ist die Therapieadhärenz eingeschränkt. Da der Verzehr von Kohlenhydraten noch dazu eine angenehme hedonische Wirkung hat, kann eine LCHF-Diät mit verringertem Genuss und Freude verbunden sein. Das wiederum könnte der Grund sein, warum eine längerfristige Einhaltung dieser Diät ein Problem darstellt [156]. Darüber hinaus ist zu bedenken, dass die meisten Patient:innen eine Kohlenhydratreduktion durch eine höhere Fettaufnahme kompensieren. Bei Nichtbeachtung der Qualität der Kohlenhydrate und Fette könnte das langfristige Risiko für kardiovaskuläre Erkrankungen steigen. Weiters kann diese Einschränkung der Lebensmittelauswahl mit einem Risiko einer unzureichenden Nährstoffversorgung verbunden sein sowie sich negativ auf die Aufnahme von Ballaststoffen auswirken [156–158].

Pauschal gesehen dürfte also immer noch die Gesamtkalorienaufnahme der beste Prädiktor für Gewichtsverlust und Verbesserung der glykämischen Stoffwechsellage sein und nicht eine alleinige Reduktion der Kohlenhydrate [146]. Eine kohlenhydratarme Ernährung kann für Personen mit Diabetes und Übergewicht sowie Adipositas eine kurz wirksame (bis zu 6 Monaten) Möglichkeit zur Verbesserung der glykämischen Kontrolle und der Triglyzeride darstellen und sollte nur unter medizinischer und diätologischer Begleitung erfolgen. Einerseits gilt es zu beachten, dass bei einer Umstellung auf eine kohlenhydratärmere Ernährung das Hypoglykämierisiko steigt und somit bei Bedarf die Diabetesmedikation angepasst werden muss, andererseits ist es durch die Einschränkung der Lebensmittelauswahl besonders von Bedeutung ernährungstherapeutisch zu unterstützen, um eine ausreichende Ballaststoffzufuhr in Form von Gemüse, Hülsenfrüchten, Vollkornprodukten und Obst sicherzustellen und die Zufuhr von gesättigten Fettsäuren gering zu halten [155].

### 6.2 Mediterrane Ernährung

Die traditionelle mediterrane Ernährung ist ebenfalls als kohlenhydratreduzierte Ernährungsform („moderate-carb“) einzustufen, welche von der ADA und EASD verglichen zu „low-carb“ als übergeordnet eingestuft wird [159]. Der in der Literatur beschriebene Terminus „mediterrane Diät“ impliziert durch das Wort Diät einen streng abgestimmten Mahlzeitenplan, vielmehr sollte es als eine Art Lebensweise verstanden werden, bei der der Genuss von saisonalen und frischen Mahlzeiten im Vordergrund steht und die den Verzehr von mehr Gemüse, Hülsenfrüchten, Nüssen, Samen, frischem Obst, vollkornreichen Lebensmitteln, Olivenöl und Fisch sowie moderaten Konsum von Joghurt, Käse und Eiern und wenig rotem Fleisch vorsieht. Nach derzeitigem Stand der Wissenschaft zeigt die traditionelle mediterrane Ernährungsweise die besseren Erfolge bezüglich der Nüchternblutglukose und des Lipidprofils. Durch ihre positiven Auswirkungen auf Gewichtsreduktion, HbA_1c_ und Blutdruck zählt die mediterrane Ernährung neben „low-fat“ und „low-carb“ zu den 3 idealen Ernährungsweisen [160–163]. Weitere Untersuchungen werden benötigt, um den Stellenwert dieser Ernährungsform in der Diabetestherapie zu erheben.

### 6.3 Intervallfasten

Das immer größer werdende Interesse für intermittierendes Fasten wirft auch die Frage auf, ob dies Vorteile für Menschen mit Diabetes bringen kann. Fasten bedeutet, eine gewisse Zeitperiode auf Lebensmittel, Getränke oder beides zu verzichten, und hat oftmals religiöse oder spirituelle Hintergründe. Beim Fasten gilt als Ansatzpunkt die Mahlzeitenhäufigkeit zur Gewichtsreduktion und Stoffwechselverbesserung und nicht die qualitative Optimierung der Ernährung. Je nach Art des Fastens werden Zeitangaben vorgegeben, wann Essen erlaubt ist und wann nicht, ohne Angabe von genauen Ernährungsempfehlungen. Dies lässt Fasten auf den ersten Blick unkompliziert und einfach erscheinen. Verbote gibt es meistens nicht, und der Verzicht beschränkt sich immer nur auf ein paar Stunden oder Tage in der Woche – Wechselfasten, 5:2-Fasten oder zeitlich begrenztes Fasten wie 16:8. Das mag die Compliance im Vergleich zu anderen Diäten erhöhen. Jedoch ist oft nicht klar, was in den erlaubten Essphasen alles verzehrt werden darf, da eine Ernährungsumstellung nicht im Fokus liegt. Das kann wiederum zu unkontrollierten Schlemmereien führen. Eine ausgewogene, kalorienreduzierte Ernährung wäre nach wie vor das Ziel einer Gewichtsreduktion.

Beobachtungsstudien im Rahmen des Ramadans sehen bei Gesunden nur geringe und vorübergehende metabolische Veränderungen [164–166]. Die Evidenz im Hinblick auf Vorteile einer geringeren Mahlzeitenfrequenz zugunsten einer Verbesserung auf Körpergewicht, Fettmasse und Taillenumfang ist gering [164].

In Metaanalysen zum Intervallfasten finden sich keine Vorteile des Intervallfastens gegenüber einer kontinuierlichen Kalorienrestriktion. Verglichen mit einer unveränderten Kontrolldiät kommt es zu einer signifikant höheren Reduktion von Körpergewicht, Taillenumfang, Blutdruck und Triglyzeriden, nicht aber von LDL-Cholesterin, Nüchternglukose oder HbA_1c_ [167–169].

Kleinere randomisierte kontrollierte Studien mit Personen mit Diabetes konnten zeigen, dass intermittierendes Fasten im Vergleich zu Nicht-Fasten, entweder an aufeinanderfolgenden Tagen oder durch Fasten von 16 h oder mehr, zu einem Gewichtsverlust führen kann, allerdings zu keiner Verbesserung des HbA_1c_ [170]. Eine aktuelle Übersichtsarbeit zeigt eine kurzfristige Änderung der Glukoseparameter, u. a. eine Verbesserung der Insulinsensitivität [171]. Ein erhöhtes Hypoglykämierisiko sei zudem nicht außer Acht zu lassen. Generell liegt derzeit aber nur eine beschränkte Anzahl an qualitativ guten RCTs mit Personen mit Diabetes vor. Zahlreiche der dem Intervallfasten zugeschriebenen Effekte sind auf die damit verbundene Kalorienrestriktion zurückzuführen. Die Vielfalt der Fastenschemata erschwert formale Empfehlungen. Für den Einsatz in der Praxis ist eine individualisierte Risiko-Nutzen-Abwägung nötig [171–174]. Ein vorgegebenes Zeitfenster kann möglicherweise manche Personen unterstützen, ihre Stoffwechselziele zu erreichen. Allerdings ist zu berücksichtigen, dass es eine Herausforderung sein kann, innerhalb eines 8‑h-Zeitfensters die empfohlene Ballaststoffzufuhr von mindestens 30 g pro Tag sowie eine adäquate Proteinzufuhr sicherzustellen.

An den Fasttagen können zudem Nebenwirkungen auftreten, da es zu einem Abfall des Blutzuckerspiegels kommt. Mögliche Nebenwirkungen sind Schwindel, Schlafstörung, Mundgeruch, Kopfschmerzen, Konzentrationsschwäche, vermehrtes Kälteempfinden und Hungergefühl. Fasten ist für Kinder, Jugendliche, Schwangere, Stillende, Senior:innen mit Erkrankungen, Menschen mit Essstörungen oder chronisch Erkrankte nicht empfehlenswert.

### 6.4 Formuladiäten

Der Einsatz von „Formuladiäten“ mit niedrigem („low calory diet“ [LCD]; 800–1200 kcal/Tag), sehr niedrigem („very low calory diet“ [VLCD]; < 800 kcal/Tag) Kaloriengehalt oder sehr niedrigem Kalorien- und Kohlenhydratgehalt („very low calory ketogenic diet“ [VLCKD];  800 kcal/Tag,  50 g Kohlenhydrate/Tag, 1–1,5 g Protein/kg Körpergewicht/Tag) kann in bestimmten klinischen Situationen angebracht sein. Optimalerweise sollten Formuladiäten eingebettet in ein strukturiertes Gewichtsmanagementprogramm sein. Kontraindikationen für eine VLCD bzw. VLCKD sind schwangere oder stillende Frauen, ältere Menschen, Kinder und Jugendliche mit Typ-1-Diabetes, Menschen mit T2DM mit β‑Zell-Versagen, Einnahme von SGLT-2‑I, Patient:innen mit fortgeschrittener Herz‑, Leber‑, Nieren- und respiratorischer Insuffizienz, instabile Angina pectoris oder Myokardinfarkt bzw. Schlaganfall in den letzten 12 Monaten, Herzrhythmusstörungen, Essstörung oder psychiatrische Erkrankung, Missbrauch/Abhängigkeit von Alkohol oder Drogen und schwere Infektionen. Der Einsatz einer VLCD/VLCKD sollte zeitlich auf maximal 12 Wochen begrenzt werden. Für die sichere Anwendung ist eine vorherige medizinische Untersuchung zum Ausschluss von Risiken und Kontraindikationen dringend empfehlenswert. Wegen potenzieller Risiken und der notwendigen Anpassung von Medikamenten bei Begleiterkrankungen ist eine durchgehende medizinische Betreuung erforderlich.

VLCDs gehen mit einer stark hypokalorischen Ernährung einher und fördern besonders bei Personen mit Übergewicht oder Adipositas und T2DM einen raschen Gewichtsverlust. Bei einer Formuladiät wird mindestens eine Mahlzeit am Tag durch ein kalorienreduziertes Produkt in Form von industriell hergestellten Shakes, Fertigdrinks, Riegeln etc. ersetzt. Dieser wiederum resultiert in den meisten Fällen in einer signifikanten Verbesserung des Glukose- und Fettstoffwechsels [175, 176].

Formuladiäten können initial als erste Unterstützung dienen und sollen temporär und nur unter medizinischer und ernährungstherapeutischer Begleitung zum Einsatz kommen. Eine Anpassung der Diabetesmedikation ist in vielen Fällen erforderlich. Ziel sollte aber eine langfristige Umstellung zu einem gesundheitsfördernden Ernährungsverhalten sein.

## 7. Ernährungsempfehlungen bei Diabetes Typ 1

Die Ernährungsempfehlungen bei Menschen mit T1DM unterscheiden sich nicht von jenen der gesunden Allgemeinbevölkerung. Das Ziel ist eine ausgewogene und bedarfsdeckende Ernährung unter Erreichung nahe-normoglykämischer Werte. Bei bestehendem Übergewicht empfiehlt sich eine hypokalorische Ernährung unter diätologischer Begleitung zur langfristigen und nachhaltigen Gewichtsreduktion [177, 178].

Menschen mit prandialer Insulintherapie im Rahmen einer funktionellen Insulintherapie (FIT-Therapie) bzw. Insulinpumpentherapie (kontinuierliche subkutane Insulininfusion [CSII]) wird für den Konsum kohlenhydrathaltiger Lebensmittel die Berechnung und in weiterer Folge Einschätzung des Gehalts an Kohlenhydraten in Gramm bzw. KE und der postprandialen Reaktion des Nahrungsmittels bzw. der Speise empfohlen. Aufgrund von technologischen Fortschritten und um eine länderübergreifende Vereinheitlichung zu ermöglichen, sollen neu diagnostizierte Patient:innen auf Gramm an Kohlenhydraten bzw. KE (1 KE = 10 g Kohlenhydrate) anstelle der bisher üblichen Broteinheiten geschult werden. Als unterstützende Instrumente für die Abschätzung des postprandialen Blutzuckerverlaufs eignen sich der GI bzw. die GL und die „Kohlenhydrat-zu-Ballaststoff-Ratio“ (KH:Bst-Ratio).

Das Wissen über die postprandiale Wirkung kohlenhydrathaltiger Speisen und Getränke sowie das Berechnen und Einschätzen von KE bzw. der Kohlenhydratmengen in Gramm sind für die Bestimmung der prandialen Insulindosierung notwendig. Da das Austesten der individuell benötigten Kohlenhydratmenge im Vordergrund steht, kann keine explizite Mengenempfehlung ausgesprochen werden [127, 177, 179]. Das Berechnen und Einschätzen der Kohlenhydratmengen einer Mahlzeit soll in strukturierten Ernährungsschulungen durch spezialisierte Diaetolog:innen erlernt werden.

Für alle Patient:innen mit einem Insulinmangeldiabetes, insbesondere einem T1DM, ist eine ketogene Diät bzw. Very-Low-Carb-Ernährungsweise nicht zu empfehlen, da das Risiko einer Ketoazidose aufgrund einer zu drastischen Insulinreduktion nicht unterschätzt werden darf. Dies kann besonders riskant sein, wenn diese Patient:innen mit SGLT2-Hemmern behandelt werden. Es sind weitere Studien mit einer größeren Studienpopulation sowie einer längeren Dauer notwendig, um exakte Empfehlungen geben zu können.

Eine individuelle Berücksichtigung der Krankheit inklusive Begleit- und Folgeerkrankungen, der Präferenzen und des Lebensstils des Patienten sind notwendig, um eine geeignete Ernährungsform langfristig umsetzen zu können [4, 127, 179].

## Abkürzungen

ADI: Acceptable daily intake
ALA: Alpha-Linolensäure
BMI: Body-Mass-Index
CSII: Kontinuierliche subkutane Insulininfusion
DHA: Docosahexaensäure
DSMES: Diabetesselbstmanagement
E%: Energieprozent
EPA: Eicosapentaensäure
FIT-Therapie: Funktionelle Insulintherapie
GI: Glykämischer Index
GL: Glykämische Last
HDL: High-density-Lipoprotein
KE: Kohlenhydrateinheiten
KG: Körpergewicht
KHK: Koronare Herzerkrankung
*LCHF*: Low-carbohydrate, high-fat
*LDL*: Low-density-Lipoprotein
*PUFA*: Mehrfach ungesättigte Fettsäuren
T1DM: Diabetes mellitus Typ 1
*T2DM*: Diabetes mellitus Typ 2
VLCD: Very low calory diet
VLCKD: Very low calory ketogenic diet
WHO: World Health Organization
